# A Redox-Switchable Colorimetric Probe for “Naked-Eye” Detection of Hypochlorous Acid and Glutathione

**DOI:** 10.3390/molecules24132455

**Published:** 2019-07-04

**Authors:** Qian Han, Fang Zhou, Yue Wang, Huan Feng, Qingtao Meng, Zhiqiang Zhang, Run Zhang

**Affiliations:** 1 School of Chemical Engineering, University of Science and Technology Liaoning, Anshan, Liaoning 114051, China; 2 School of Chemistry and Life Science, Anshan Normal University, Anshan 114007, China; 3 Australian Institute for Bioengineering and Nanotechnology, The University of Queensland, Brisbane 4072, Australia

**Keywords:** redox-switchable, hypochlorous acid, glutathione, colorimetric, detection, water samples

## Abstract

We report the development of a new colorimetric probe (**L-ol**) for investigations of the redox process regulated by hypochlorous acid (HOCl) and glutathione (GSH). The HOCl/GSH redox-switching cycle process was investigated in detail by UV-vis absorption spectroscopy, colorimetric analysis assay and high-resolution mass spectrometry (HRMS). The switchable absorbance responses were attributed to the HOCl-induced oxidation of the *p*-methoxyphenol unit to the benzoquinone derivative (**L-one**) and sequential reduction of **L-one** to hydroquinone (**L-ol’**) by GSH. In phosphate-buffered saline (PBS) buffer, the absorbance of **L-ol** at 619 nm underwent a remarkable bathochromic-shift, accompanied by a color change from pale yellow to blue in the presence of HOCl. With further addition of GSH, the absorbance of **L-one** exclusively recovered to the original level. Meanwhile, the blue-colored solution returned to the naive pale yellow color in the presence of GSH. The detection limits for HOCl and GSH were calculated to be 6.3 and 96 nM according to the IUPAC criteria. Furthermore, **L-ol**-loaded chromatography plates have been prepared and successfully applied to visualize and quantitatively analyze HOCl in several natural waters.

## 1. Introduction

The redox balance between reducing and oxidizing species is important in the regulation of a signal pathway in biosystems. The redox process is normally achieved by a series of elaborate mechanisms [1,2,3,4]. The reduction–oxidation state of the cell is primarily a consequence of the precise balance between the levels of reactive oxygen species (ROS) and antioxidant [5,6,7,8]. Among these ROS, hypochlorous acid (HOCl) is an endogenous microbicidal agent and a highly potent oxidant that is mainly generated by the catalysis of myeloperoxidase (MPO) in the presence of chloride and hydrogen peroxide under physiological conditions [9,10,11]. Owing to the strong oxidizing effect, HOCl could defend against the invasion of bacteria and regulate the life cycle of the cell [12]. Nevertheless, the excessive or misplaced production of HOCl in living systems may lead to extensive oxidative stress and oxidative damage, which can induce serious diseases such as arthritis, cardiovascular diseases, atherosclerosis, neuron degeneration, and cancer [13,14,15,16,17]. Glutathione (GSH) is the most abundant non-protein biothiol in mammalian cells, which was considered to be the main player in combating oxidative stress and regulating the redox environment of the internal cellular compartments [18,19,20,21,22,23]. Variations in GSH concentration have been linked to many pathological processes including cancer, aging, and diabetes [24,25,26,27]. It is reported that endogenous GSH was found to protect cells from the damage caused by HOCl [28]. Therefore, much effort has been focused on the development of effective detection methods for visualizing or monitoring the redox cycle between HOCl and GSH, which will be beneficial for fully understanding and treating numerous pathological diseases [29,30,31,32,33]. In comparison with other technologies, organic colorimetric probes are desirable for detecting various kinds of highly reactive species such as HOCl and GSH, owing to their high sensitivity, excellent selectivity, real-time analysis and in situ observation [34].

**Scheme 1:**
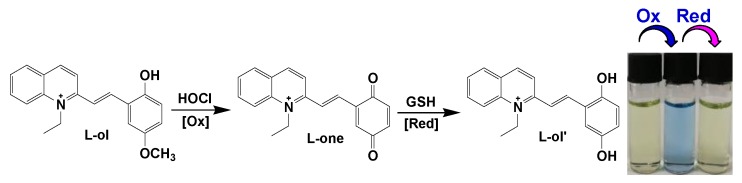
The redox-switchable behaviors of **L-ol** regulated by hypochlorous acid (HOCl) and glutathione (GSH).

Although increasing amounts of molecular probes for HOCl and GSH detection have been reported, only few of them have been applied to monitor the redox changes mediated by HOCl and antioxidants, such as GSH, simultaneously [35,36,37,38]. In this work, a novel redox-switchable molecular probe, **L-ol** was designed and synthesized by introduction of *p*-methoxyphenol as a redox response unit. It is reported that *p*-methoxyphenol can be selectively oxidized to benzoquinone by HOCl [39,40,41,42,43]. After further treatment of the obtained *p*-benzoquinone (**L-one**) by glutathione (GSH) [44], a new reduced product of **L-ol’** was obtained (Scheme 1). In addition, the redox process was accompanied by a visual pale yellow to blue to pale yellow color change, which is consistent with the UV−vis spectra. Furthermore, **L-ol**-based test plates have been successfully prepared for the visualization and quantitative analysis of HOCl in natural water.

## 2. Results and Discussion

### 2.1. Design, Synthesis and Characterization of the Molecular Probe

**Scheme 2:**
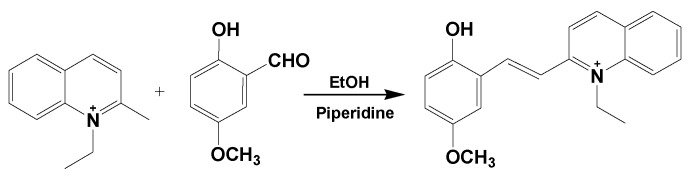
Synthetic procedure of **L-ol**.

**L-ol** was synthesized by one-step condensation reaction between 1-ethyl-2-methylquinolinium iodide and 2-hydroxy-5-methoxy benzaldehyde in the presence of a catalytic amount of piperidine (2 drops) in methanol (Scheme 2). The chemical structure of **L-ol** was characterized by ^1^H NMR, ^13^C NMR and high-resolution mass spectrometry (HRMS) (Figure S1–3). Absorbance of **L-ol** in aqueous media (dimethylformamide (DMF):phosphate-buffered saline (PBS) = 7:3, 20 mM, pH = 7.4) was not changed within 24 h (Figure S4), indicating the high optical stability of **L-ol** under the test condition.

The HOCl/GSH regulated redox-switching process was first monitored by the HRMS. As shown in Figure S3, the peak at 306.1484 was obtained as the molecular ion peak of [**L-ol**]^+^ (calcd. 306.1489). Upon addition of HOCl into the aqueous solution of **L-o**l, the peak at m/z = 306.1484 disappeared and a new molecular ion peak emerged which can be assigned to *p*-benzoquinone derivative (**L-one**) at m/z = 290.1173 (Figure S5). After further treatment of the generated **L-one** with GSH, the appearance of the peak at m/z = 292.1328 indicated the generation of the reduced product of **L-ol**’ (Figure S6). The HRMS titration analysis confirmed the proposed redox-switchable behaviors of **L-ol** regulated by HOCl and GSH (Scheme 1).

### 2.2. Oxidation of L-ol by HOCl and UV-vis Spectra Studies

**Figure 1:**
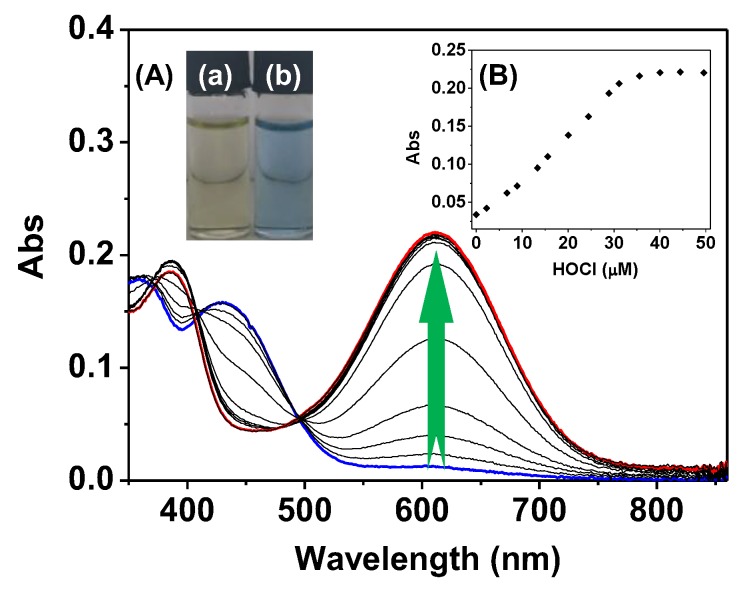
UV-vis absorption spectra of **L-ol** (10 μM) in the presence of different amounts of HOCl (0–50 μM) in phosphate-buffered saline (PBS) aqueous buffer (dimethylformamide (DMF):PBS = 7:3, 20 mM, pH = 7.4). Inset: A. Colorimetric changes of **L-ol** in the (a) absence and (b) presence of HOCl; B. Plot of the absorbance at 619 nm of **L-ol** against the concentration of HOCl.

Upon addition of increasing amounts of HOCl, UV-vis absorption spectra of **L-ol** in PBS aqueous buffer (DMF:PBS = 7:3, 20 mM, pH = 7.4) were recorded. As shown in Figure 1, free **L-ol** exhibited strong absorption band centred at 366 nm and 435 nm (log*ε*_1_ = 3.50). Upon the addition of HOCl, the two peaks decreased notably and an intense absorption band centred at 619 nm appeared (log*ε*_2_ = 4.36). The new absorption was attributed to the oxidation of the *p*-methoxyphenol to the corresponding benzoquinone derivative (**L-one**) [45]. Absorbance at 619 nm became constant when the concentration of HOCl was >40 μM (Figure 1, inset (B)). Three clear isosbestic points were found to be at 378, 404 and 496 nm, which indicated the quantitative reaction of **L-ol** with HOCl. In agreement with the obvious bathochromic-shift changes for **L-ol** after introducing HOCl, the color of the **L-ol** solution changed from pale yellow to blue (Figure 1, inset (A)). Obvious color changes of the solution suggest that **L-ol** can serve as a potential “naked-eye” indicator for HOCl detection in water samples. In addition, the absorbance at 619 nm of **L-ol** exhibited a good linear relationship with the concentration of HOCl (2.5–25.0 μM) (Figure S7). According to the IUPAC criteria (LOD = 3*σ*/k), the detection limit (LOD) was calculated to be 6.3 nM.

**Figure 2:**
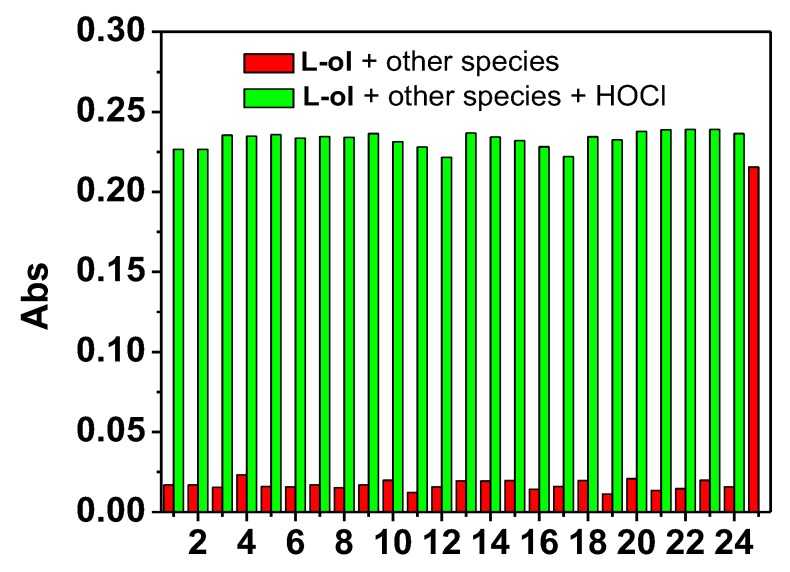
Absorption spectra responses of **L-ol** (10 μM) towards various competitive species (50 μM) in PBS aqueous buffer (DMF:PBS = 7:3, 20 mM, pH = 7.4). 1. Br^–^, 2. AcO^–^, 3. Cl^–^, 4. F^–^, 5. HSO_3_^–^, 6. HSO_4_^–^, 7. S^2–^, 8. NO_2_^–^, 9. NO_3_^–^, 10. OH^–^, 11. PO_4_^2–^, 12. SO_3_^2–^, 13. HCO_3_^–^, 14. Pi, 15. PPi, 16. HOCl, 17. ^1^O_2_, 18. ONOO^–^, 19. ·OH, 20. H_2_O_2_, 21. Cys, 22. Hcy, 23. GSH, 24. mixed competitive species, 25. HOCl.

It has been found that *p*-methoxyphenol was stable toward most other common ROS [46]. The selectivity of **L-ol** towards HOCl over other competitive ROS (^1^O_2_, ONOO^–^, •OH, H_2_O_2_), anions (Br^–^, AcO^–^, Cl^–^, F^–^, HSO_3_^–^, HSO_4_^–^, S^2–^, NO_2_^–^, NO_3_^–^, PO_4_^2–^, SO_3_^2–^, SO_4_^2–^, HCO_3_^–^, OH^–^, Pi, PPi) and biothiols (Cys, Hcy and GSH) were investigated by UV-vis absorption spectra. The change in the absorption spectra was found to be specific towards HOCl. Significant changes in the absorption spectra were not observed upon addition of the aforementioned competitive species (Figure S8). In addition, addition of 1.0 equiv. of HOCl to the above solution led to the increase of absorbance, and the increased absorbance was similar to the one of the addition of 1.0 equiv. of HOCl alone to **L-ol** solution (Figure 2). These observations indicate that the HOCl-specific responses were not disturbed by competitive species.

**Figure 3:**
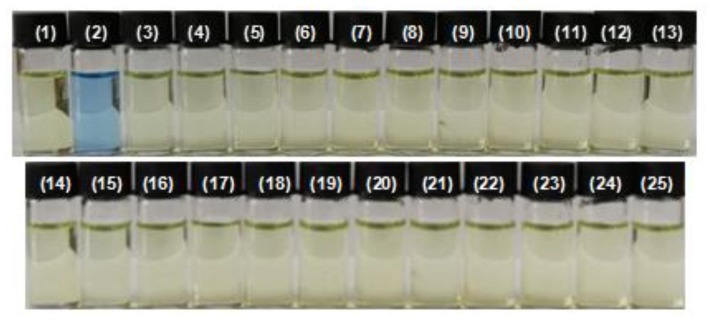
The color changes of **L-ol** (10 μM) towards various competitive species (50 μM) in PBS aqueous buffer (DMF:PBS = 7:3, 20 mM, pH = 7.4). 1. Free **L-ol**, 2. HOCl, 3. Br^–^, 4. AcO^–^, 5. Cl^–^, 6. F^–^, 7. HSO_3_^–^, 8. HSO_4_^–^, 9. S^2–^, 10. NO_2_^–^, 11. NO_3_^–^, 12. OH^–^, 13. PO_4_^2–^, 14. SO_3_^2–^, 15. HCO_3_^–^, 16. Pi, 17. PPi, 18. HOCl, 19. ^1^O_2_, 20. ONOO^–^, 21. ·OH, 22. H_2_O_2_, 23. Cys, 24. Hcy, 25. GSH.

The specificity of **L-ol** to HOCl was also confirmed by a “naked-eye” colorimetric assay. The color of **L-ol** solution specifically changed from pale yellow to blue with the titration of HOCl, while distinct color changes were not found upon addition of other biological species including ROS, anions and biomolecules (Figure 3). The results of colorimetric analysis suggest that **L-ol** can be employed as a specific probe for “naked-eye” discrimination of HOCl without any spectroscopic instrumentation.

### 2.3. Reduction of L-one by GSH and UV-vis Spectra Studies

**Figure 4:**
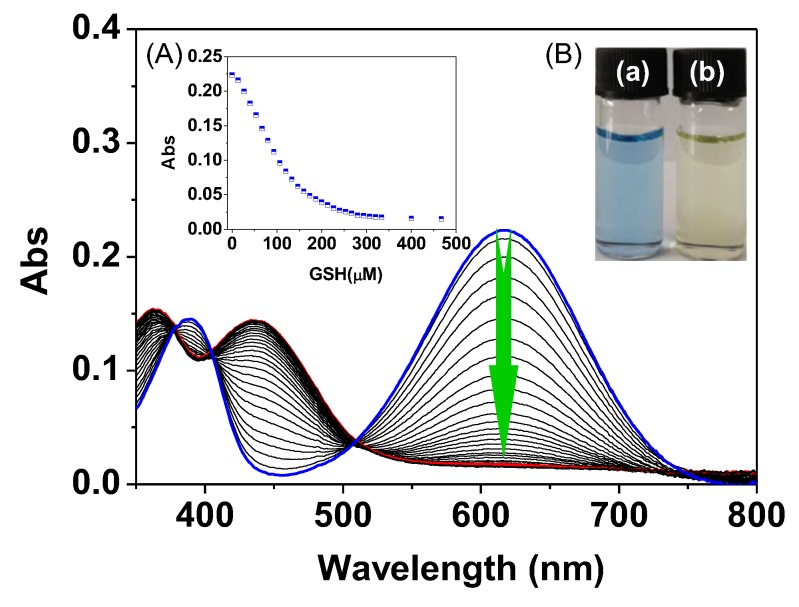
UV-vis absorption spectra of **L-one** (10 μM) in the presence of different amounts of GSH (0–500 μM) in PBS aqueous buffer (DMF:PBS = 7:3, 20 mM, pH = 7.4). Inset: (A) Plot of the absorbance at 617 nm of **L-one** against the concentration of GSH; (B) Colorimetric changes of **L-one** in the (a) absence and (b) presence of GSH.

The reduction process of the obtained **L-one** in the presence of GSH was also evaluated by recording the changes of the corresponding UV-Vis absorption spectra. After further treatment of **L-one** with GSH in phosphate buffer (20 mM, pH = 7.4), the absorption band at 619 nm was decreased gradually (log*ε*_3_ = 3.24), accompanied with the increase of the absorption band at 435 nm (Figure 4). The final absorption spectrum is almost identical to the one of that of **L-ol** under identical conditions, suggesting that the hydroquinone (**L-ol’**) was quantitatively obtained in the presence of GSH. Furthermore, the solution of **L-one** recovered to be pale yellow color (Figure 4, inset). After the GSH reduction process, the recovery of the absorption spectra and the color of the solution can be easily observed by the naked eye. Corresponding changes in UV-vis absorbance of **L-on**e are linearly proportional to the concentration of GSH in the range of 0–60 µM (Figure S9). The detection limit for GSH was calculated to be 96 nM according to the methods defined by IUPAC.

**Figure 5:**
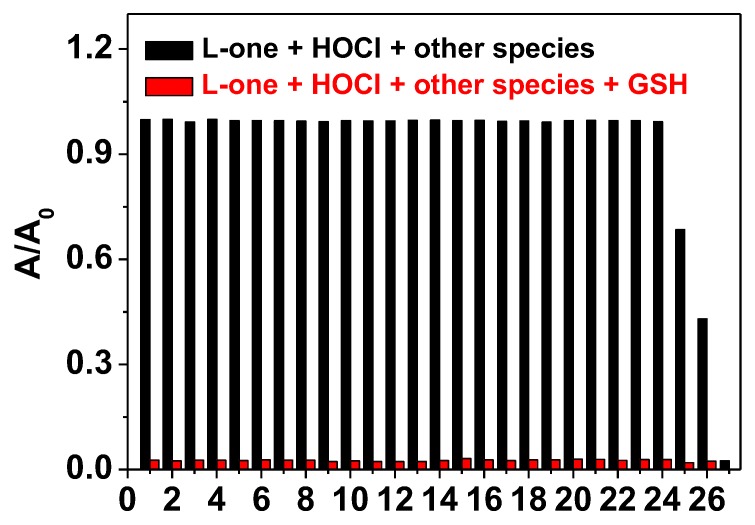
UV-vis absorption spectra of **L-one** (10 μM) in the presence of various analytes (500 μM) in PBS aqueous buffer (DMF:PBS = 7:3, 20 mM, pH = 7.4): 1. Leu, 2. His, 3. Val, 4. Tyr, 5. Gerl, 6. Try, 7. Phe, 8. Leu, 9. Thr, 10. Lys, 11. Gly, 12. Pro, 13. Ary, 14. Gln, 15. Asn, 16. Asp, 17. Ala, 18. Hcy, 19. Cys, 20. S^2–^, 21. HSO_4_^–^, 22. HSO_3_^–^, 23. SO_4_^2–^, 24. SO_3_^2–^, 25. Met, 26, Vc, 27. GSH.

To further explore the reduction specificity of **L-one** to GSH in the presence of competitive species, the absorbance responses towards several amino acids, biothiols and anions, such as Leu, His, Val, Tyr, Gerl, Try, Phe, Leu, Thr, Lys, Gly, Pro, Ary, Gln, Asn, Asp, Ala, Met, Hcy, Cys, S^2–^, HSO_4_^–^, HSO_3_^–^, SO_4_^2–^, SO_3_^2–^ and ascorbic acid (Vc) were investigated. As shown in Figure S10, the absorption peak of **L-one** centered at 619 nm remarkably decreased upon addition of GSH, while only a small decrease of the absorbance of **L-one** was observed upon addition of Met and Vc. An obvious decrease of the UV-vis absorption was not observed in the presence of other competitive species. In addition, the absorbance response of **L-one** towards GSH in the presence of other interfering species is nearly identical to that in the absence of these species (Figure 5). The results illustrated that **L-one** does have a specific response toward GSH over other common interfering species. The GSH-specific reduction process of **L-one** was also tested by colorimetric assay with a verity of competitive species. Upon addition of GSH, the solution color of **L-one** recovered to pale yellow from blue, while negligible color changes were found for the **L-one** solution in the presence of Met and Vc. The specific response of GSH over Met and Vc is attributed to the powerful reducibility of GSH [35]. Moreover, no obvious solution color changes were noticed upon addition of other competitive amino acids, biothiols and anions, further indicating that **L-one** was specifically reduced by GSH (Figure S11).

**Figure 6:**
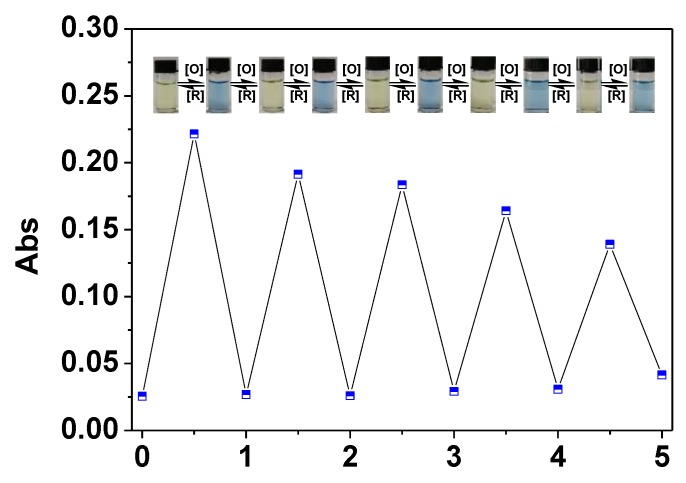
UV-vis absorption spectra changes at 619 nm of **L-ol** (10 μM) in PBS aqueous buffer (DMF:PBS = 7:3, 20 mM, pH = 7.4) upon the alternate addition of HOCl/GSH with several concentration ratios (0:0, 20:0, 20:50, 40:50, 40:100, 60:100, 80:100, 80:150, 100:150, 120:150 and 120:200 μM, respectively). [O], oxidation; [R], reduction.

**Figure 7:**
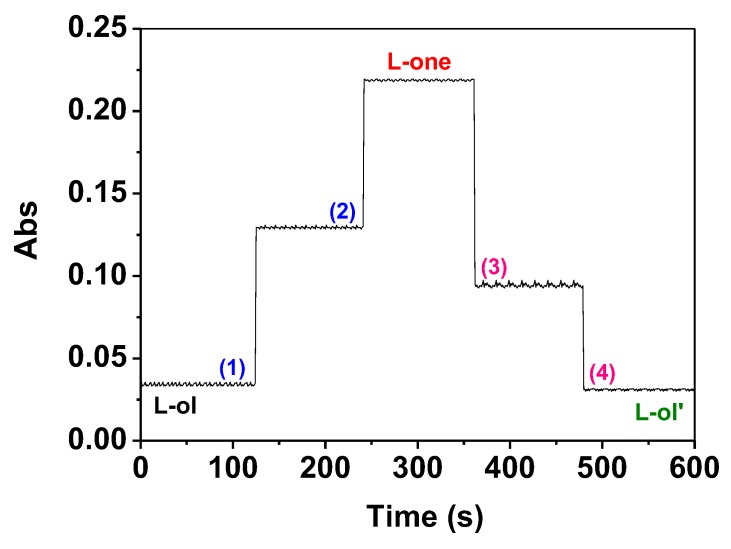
Time course UV-vis absorption response of **L-ol** (10 μM) to (1) 20 μM, (2) 50 μM HOCl, and *in-situ* formed **L-one** to (3) 100 μM (4) 500 μM GSH in PBS aqueous buffer (DMSO: H_2_O = 9:1, 20 mM, pH = 7.4).

Reversible regulation of the redox-switching process was then examined by alternate addition of HOCl and GSH into the solution of **L-ol** in PBS aqueous buffer. As shown in Figure 6, the absorbance of **L-ol** gives rise to a switchable change at 619 nm. Such a reversible absorbance increase/decrease of **L-o**l could be repeated at least 6 times. Simultaneously, the redox process was accompanied by a visual pale yellow to blue to pale yellow color change cycle (Figure 6, inset), which is consistent with the absorbance response. The time-dependent redox cycle of **L-ol** regulated by HOCl/GSH was then investigated. As shown in Figure 7, upon the addition of HOCl, absorbance at 619 nm of **L-ol** was rapidly increased and reached the maximum value within 1 s, and then kept at a steady level. When another amount of HOCl was added, absorbance was increased rapidly again and reached another plateau rapidly. In addition, the reduction process of **L-one** towards GSH was also completed within 1 s. The results indicate that **L-ol** could be used to monitor the HOCl/GSH regulated redox-switching process in real-time.

**Figure 8:**
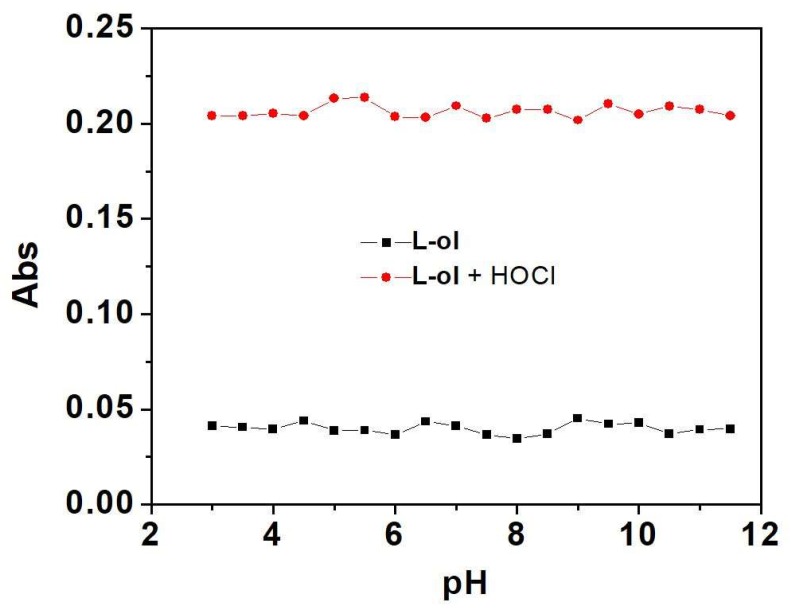
Effect of pH on the absorbance of **L-ol** (10 μM) at 619 nm in the absence and presence of HOCl (50 μM).

The pH-dependent absorbance behavior of **L-ol** was also explored. As shown in Figure 8, in the absence of HOCl, **L-ol** emerges weak absorbance of 619 nm over the whole pH range investigated. In the assessment of pH responses of **L-ol** in the presence of HOCl, it was found that the absorbance of **L-ol** at 619 nm was pH independent in the range of 3.0–11.5. The results indicated that **L-ol** feature high tolerance in a wide pH range.

### 2.4. Application of L-ol-Loaded Chromatography Plates in the Quantitative Analysis of HOCl in Natural Water Samples

**Figure 9:**
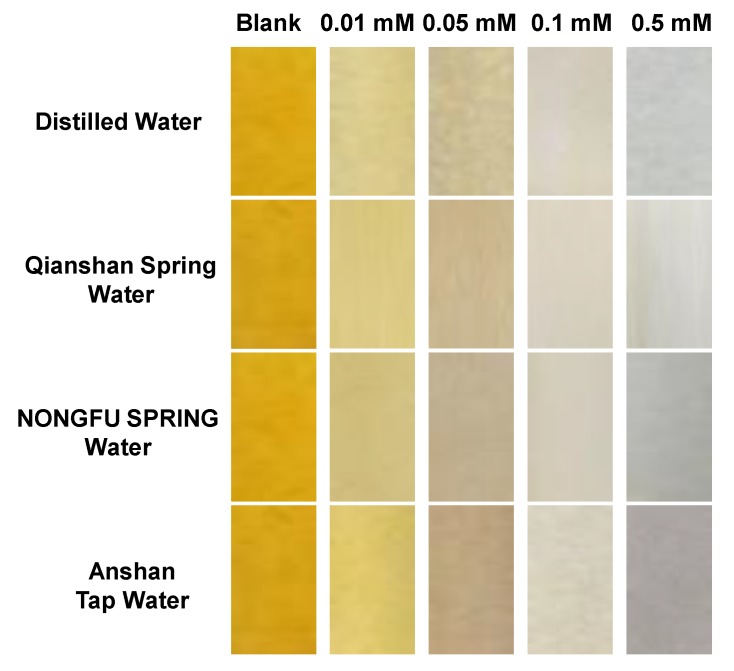
Color responses of **L-ol**-coated chromatography plates towards different concentrations of HOCl (0–0.5 mM) in several natural water samples.

HOCl is widely used in our daily life, such as in the disinfection of drinking water. High concentrations of HOCl used in water treatments is a potential health threat to human and animal lives [47]. Therefore, it is of great significance to monitor the level of HOCl in environmental samples in real time. Accordingly, **L-ol**-coated chromatography plates were prepared for the “naked-eye” quantitative analysis of HOCl in several water samples. As shown in Figure 9, after being exposed to different concentrations of HOCl (0–0.5 mM) in distilled water, the **L-ol**-coated chromatography plates presented immediately a color change from pale yellow to blue under sunlight. Next, the performances of **L-ol**-coated test plates for the detection of HOCl in potential practical water samples, such as Qianshan Spring water, NONGFU SPRING water and Anshan tap water. It has been found that similar color changes were observed for the water samples containing various amounts of HOCl. Clearly, the test plates can detect HOCl in an aqueous solution at a low limit of about 0.01 mM, indicating the potential application for quantitative analysis of HOCl levels without any spectroscopic instrumentation.

## 3. Experimental

### 3.1. Reagents and Materials

1-Ethyl-2-methylquinolinium iodide was prepared according to our reported methods [48,49]. 2-Methylquinoline, iodoethane, 2-hydroxy-5-methoxy benzaldehyde were purchased from Aladdin reagent Co. (Shanghai, China). Anions (sodium salts), sodium hypochlorite (NaOCl), 3-morpholinosydnonimine (SIN-1) (ONOO^−^ donor) were purchased from Alfa Aesar (Zhengzhou, China). The chromatography plates were purchased from commercial source with specifications of 25 cm × 75 cm^2^, 0.20–0.25 mm thickness. Unless otherwise stated, solvents and reagents were of analytical grade from commercial suppliers and were used without further purification. Deionized water was used throughout.

### 3.2. Apparatus

^1^H-NMR and ^13^C-NMR spectra were recorded with an AVANCE600MHZ spectrometer (Bruker, Fällanden, Switzerland) with chemical shifts reported as *ppm* (in DMSO, TMS as internal standard). High resolution mass spectra (HR MS) were recorded on an Agilent 6530 QTOF spectrometer (Singapore). Absorption spectra were measured with a Perkin Elmer Lambda 900 UV/VIS/NIR spectrophotometer (Perkin Elmer, Waltham, MA, USA). Quartz cuvettes with a 1 cm path length and 3 mL volume were involved in fluorescence and UV-vis spectrum measurements. The pH was recorded by OHAUS ST3100 digital pH-meter (Changzhou, China).

### 3.3. Synthesis and Characterization of L-ol

1-Ethyl-2-methylquinolinium iodide (0.299 g, 1 mmol) and 2-hydroxy-5-methoxy benzaldehyde (0.152 g, 1 mmol) were dissolved in moderate amounts of absolute methanol, respectively. The mixture was then refluxed at 80 ^o^C for 8 h to form a dark green precipitate. The crude product was immediately filtered, and washed with cooled methanol to obtain **L-ol** in 73.4% yield. ^1^H NMR (DMSO-*d*_6_, 600 MHz), δ(*ppm*) 10.29 (s, 1H), 9.04 (d, *J* = 13.44 Hz, 1H), 8.54-8.59 (2H), 8.39 (t, *J* = 11.16 Hz, 1H), 8.25-8.29 (d, *J* = 23.64 Hz, 1H), 8.20 (t, *J* = 11.19 Hz, 1H), 7.95 (d, *J* = 8.4, 1H), 7.93 (t, *J* = 3.66 Hz, 1H), 7.46 (d, *J* = 4.26 Hz, 1H), 7.02 (d, *J* = 13.32 Hz, 1H), 6.94 (d, *J* = 13.32, 1H), 5.12 (q, *J* = 10.74 Hz, 2H), 3.79 (s, 3H),1.60 (t, *J* = 10.65 Hz, 3H). ^13^C NMR (DMSO-*d*_6_, 125 MHz), *δ* (*pp*m) 155.1, 152.9, 151.4, 143.1, 137.5, 134.4, 129.7, 126.3, 123.8, 121.2, 120.5, 119.8, 118.1, 117.3, 115.1, 112.7, 54.9, 46.0, 18.0, 13.1. HRMS-API (positive mode, m/z) for [**L-ol**]^+^: calcd 306.1489, found: 306.1484.

### 3.4. Preparation of Stock Solutions of Probes and Analyte

Stock solution of **L-ol** at the concentration of 0.5 mM was prepared by dissolving certain amounts of probes in DMF. For the detection of HOCl in buffer, the stock solution of **L-ol** was diluted into 20 mM PBS of pH 7.4 (DMF:H_2_O = 7:3, v/v) for all spectrometric measurements. For the absorption response of **L-ol** to HOCl and **L-one** to GSH, the spectra were recorded after mixing the analyte and probe for 5 min at room temperature. Solutions of a series of anions and biothiols (20 mM) were freshly prepared by dissolving corresponding chemicals in deionized water. The ROS species were prepared in deionized water according to previous literature [50,51,52,53]. A stock solution of HOCl was prepared by diluting the commercial NaOCl solution and stored. Hydroxylradical (·OH) was generated in the Fenton system from ferrous ammonium sulfate and hydrogen peroxide. Superoxide anion radical (O_2_^−^) was generated from the xanthine-xanthine oxidase system. Singlet oxygen (^1^O_2_) was generated from the Na_2_MoO_4_-H_2_O_2_ system in 0.05 M carbonate buffer of pH 10.5. ONOO^−^ was obtained by using SIN-1 as a donor. Hydrogen peroxide (H_2_O_2_) was diluted immediately from a stabilized 30% solution, and was assayed using its molar absorption coefficient of 43.6 M^−1^ cm^−1^ at 240 nm.

### 3.5. “Naked-Eye” Analysis of HOCl in Natural Water Samples by L-ol-Based Chromatography Plates

The silica gel plates (25 × 75 mm) were immersed into 50 mL dichloromethane solution of **L-ol** (0.5 mM) for 10 min. Then, the **L-ol**-coated silica gel plates were dried in air for further measurements.

Water samples were from four different sources: distilled water (DW), tap water (TW) from Anshan City (Liaoning Province, China), commercial drinking water (CW) (NONGFU Spring, China) and spring water (SW) from the Qianshan Mountain (Liaoning Province, China). Different stock solutions of HOCl were prepared using the above water samples. For “naked-eye” analysis of HOCl in the above natural water samples, the **L-ol**-coated chromatography plates were immersed in the HOCl aqueous solution with different concentrations for several seconds then dried in air. Then, the color changes of **L-ol**-coated chromatography plates were recorded under visible light.

## 4. Conclusions

In summary, a novel *p*-methoxyphenol-based reversible colorimetric probe, **L-ol**, has been developed to selectively detect the HOCl/GSH redox cycle in aqueous solution. By the reversible redox reaction, **L-ol** exhibited a switchable absorbance response along with a pale yellow to blue to pale yellow color change cycle. The redox-switchable responses of **L-ol** in ROS/GSH couple with excellent reversibility. The desirable features of **L-ol** for the detection of HOCl in aqueous solutions, such as its high sensitivity and selectivity, observable color response and reliability at physiological pH enabled its application in the detection of HOCl in natural water samples. The easy-to-prepare **L-ol**-loaded chromatography plates have been successfully prepared. The preliminary investigations in natural water samples offer direct and immediate detection of HOCl in complex media in real time.

## Supplementary Materials

The following are available online, Figure S1-3: NMR and HRMS characterizations of **L-ol**, Figure S4: The stability of **L-ol**, Figure S5,6: HRMS of **L-ol** sequential addition of HOCl and GSH, Figure S7: Linear relationship of **L-ol** with the concentration of HOCl. Figure S8: Absorption spectra of **L-ol** upon addition of various anions, Figure S9: Linear relationship of **L-one** with the concentration of GSH, Figure S10: UV-vis absorption spectra of **L-one** in the presence of various analytes, Figure S11: The color changes of **L-one** towards various competitive species.
