# Supplementary material for: A Redox-Switchable Colorimetric Probe for “Naked-Eye” Detection of Hypochlorous Acid and Glutathione
Source: Molecules. 2019 Jul 4;24(13):2455. doi: 10.3390/molecules24132455 (PMC6651190; doi:10.3390/molecules24132455)
Supplement: Supplementary file 1 [file molecules-24-02455-s001.pdf]

Supplementary Materials

A redox-switchable colorimetric probe for "naked-eye" detection of hypochlorous acid and glutathione

Qian Han,‡[1,3] Fang Zhou,‡[1] Yue Wang,[1] Huan Feng,[1] Qingtao Meng,*[1] Zhiqiang Zhang,*[1] and Run Zhang,*[1, 2]

[1] School of Chemical Engineering, University of Science and Technology Liaoning, Anshan, Liaoning, 114051, P. R. China.

E-mail: qtmeng@ustl.edu.cn; zhangzihqiang@ustl.edu.cn.Tel.: +86-412-5929627

[2] Australian Institute for Bioengineering and Nanotechnology, The University of Queensland, Brisbane, 4072, Australia.

Email: r.zhang@uq.edu.au; Tel.: + 61 7 3346 3806, Fax: + 61 7 3346 3978

[3] School of Chemistry and Life Science, Anshan Normal University, Anshan 114007, P. R. China.

‡ These authors contributed equally to this work and they should be regarded as co-first authors.

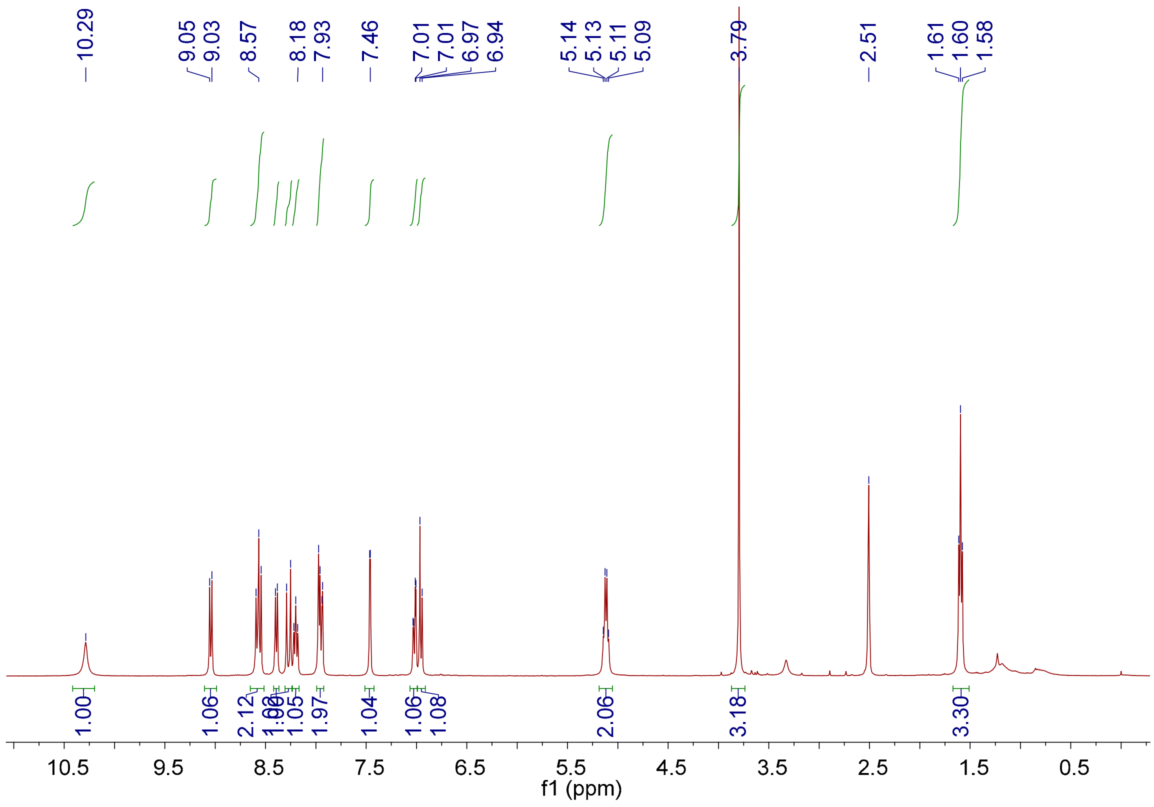

Figure S1. ^{1}H NMR of **L-ol** (DMSO-d_6).

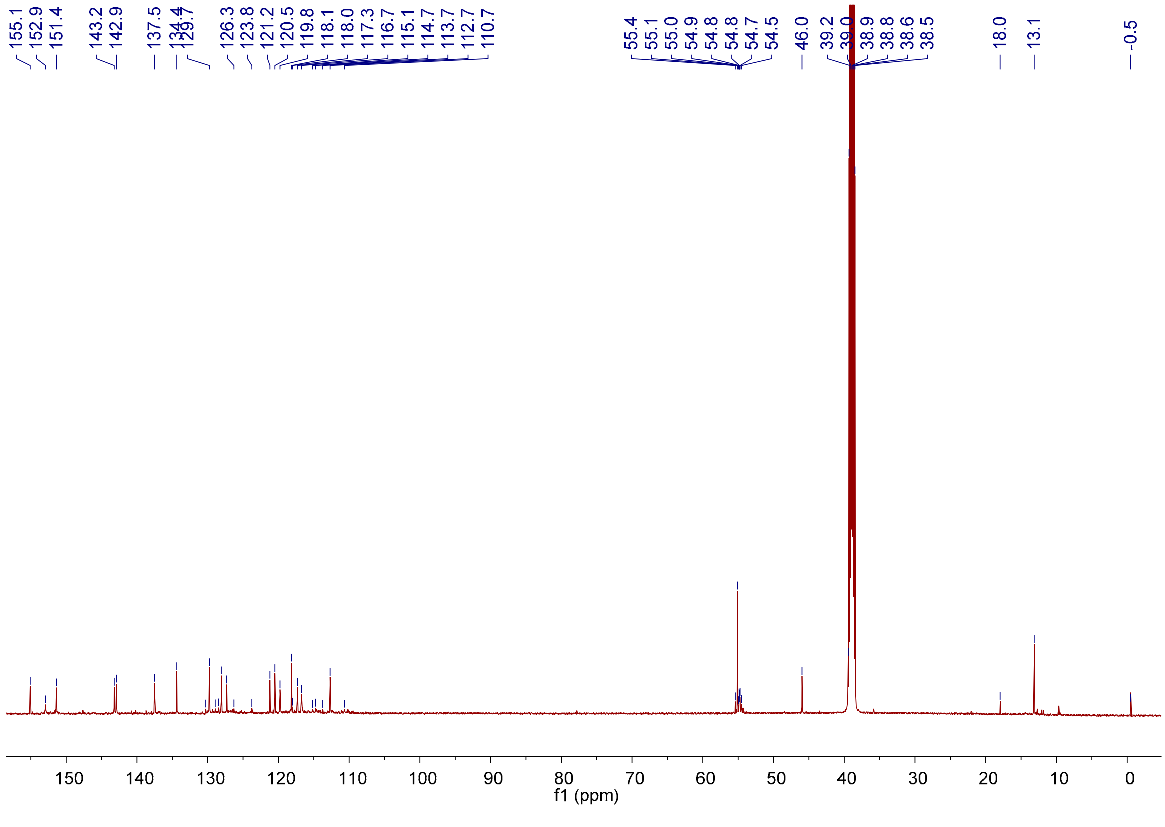

Figure S2. ^{13}C NMR of **L-ol** (DMSO-d_6).

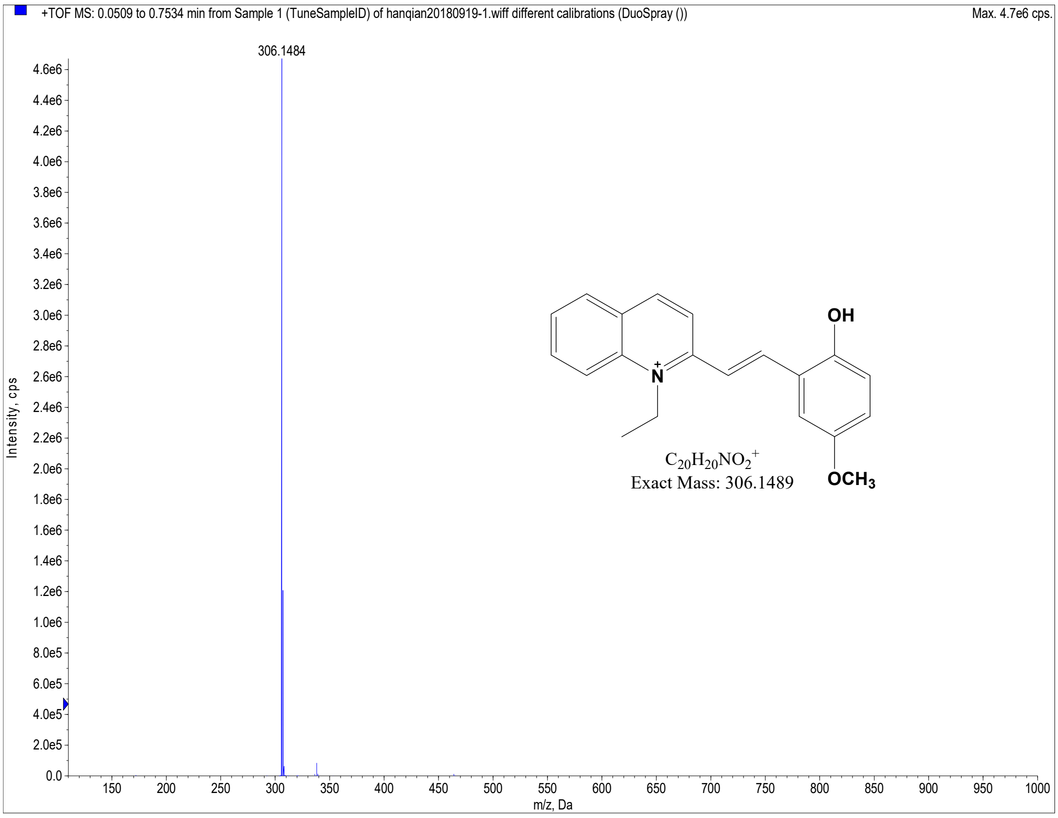

Figure S3. HRMS of **L-ol**.

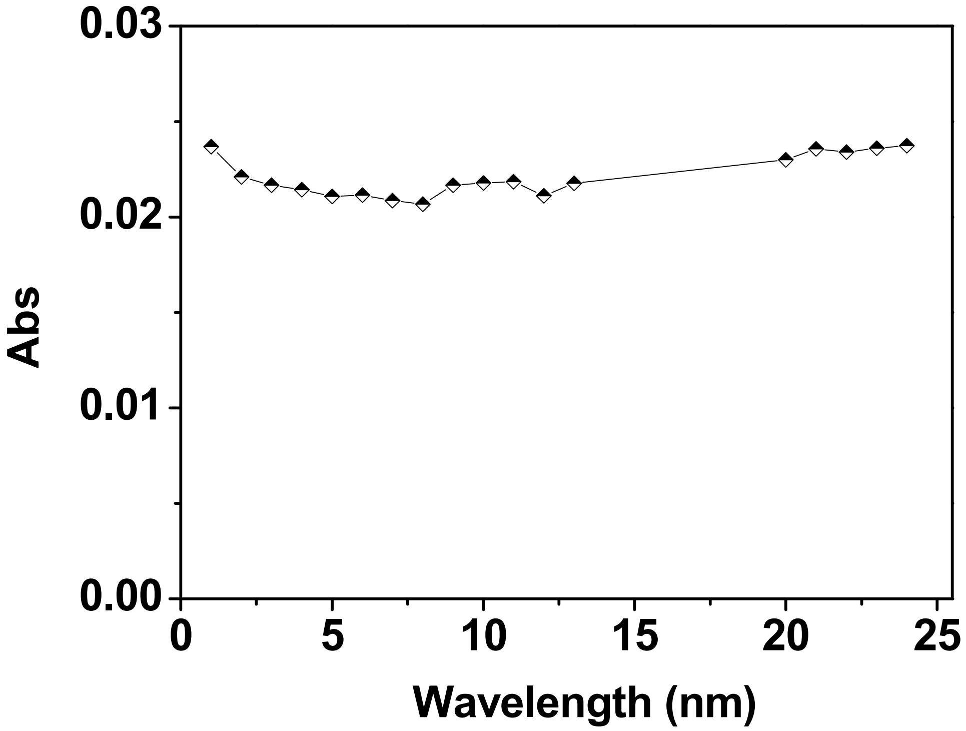

Figure S4. Absorbance at 619 nm of **L-ol** (10 μM) at different time in PBS aqueous buffer (DMF: PBS=7:3, 20 mM, pH=7.4).

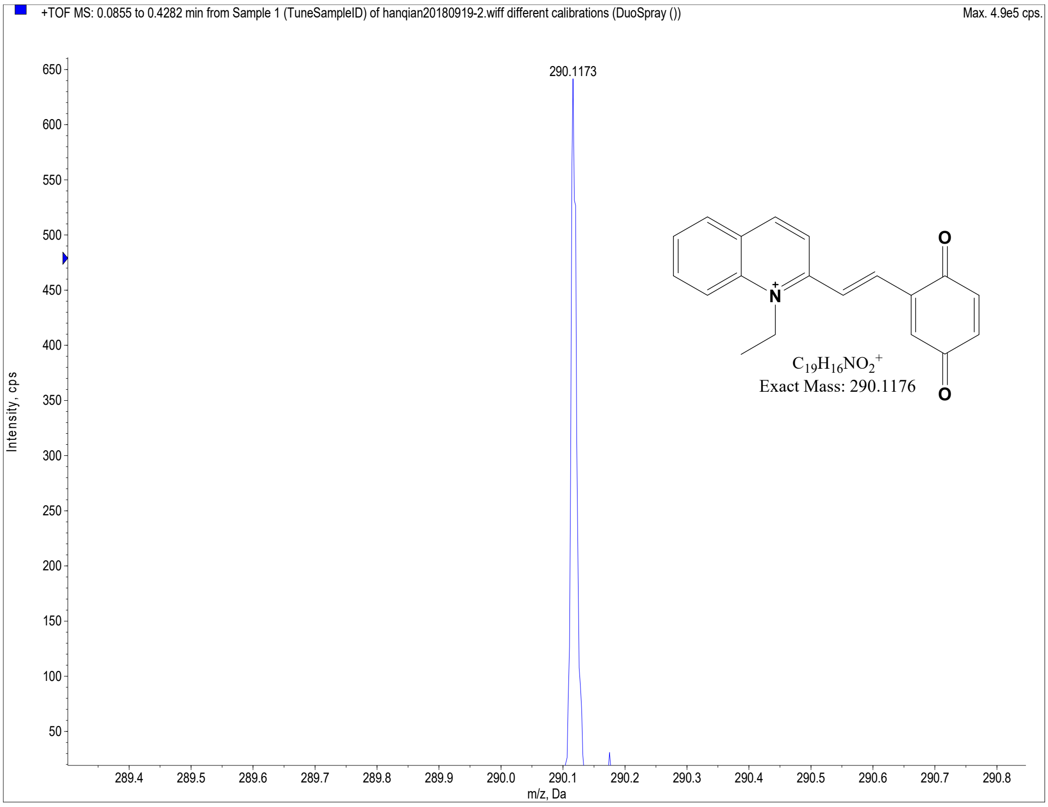

Figure S5. HRMS of **L-ol** in the presence of HOCl.

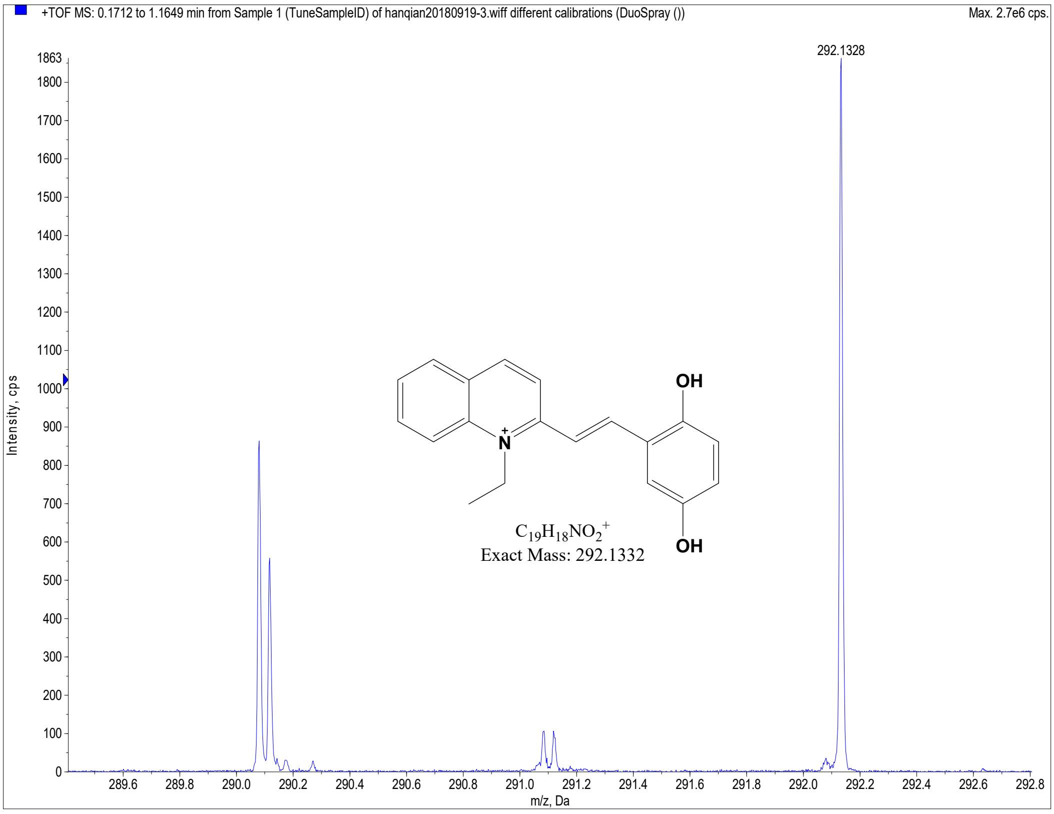

Figure S6. HRMS of HOCl pre-treated **L-ol** in the presence of GSH.

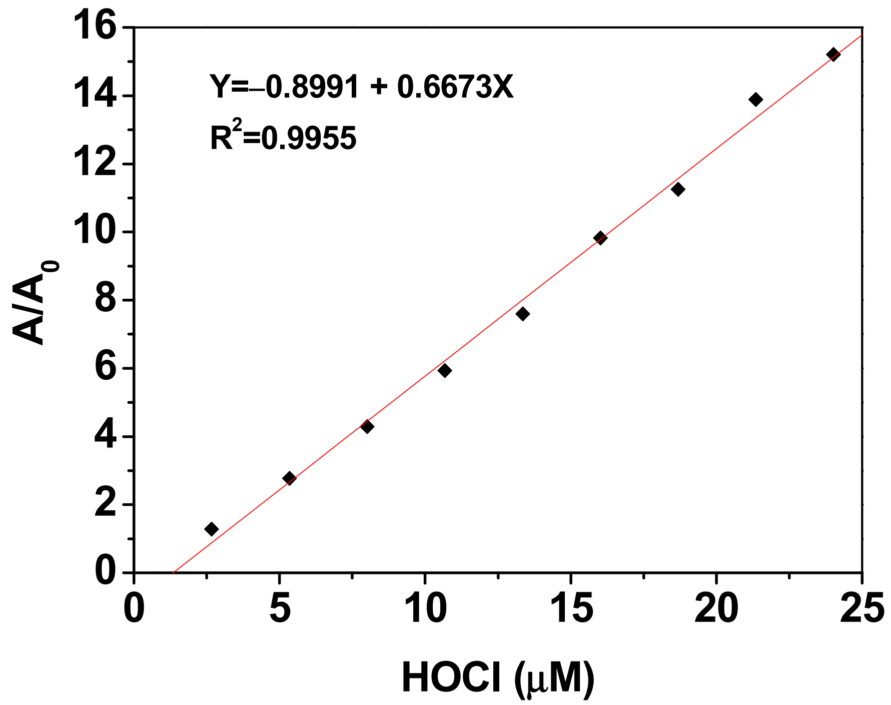

Figure S7. UV-vis absorption spectra changes at 619 nm of **L-ol** (2 μM) as a function of HOCl concentration (2.5–25.0 μM).

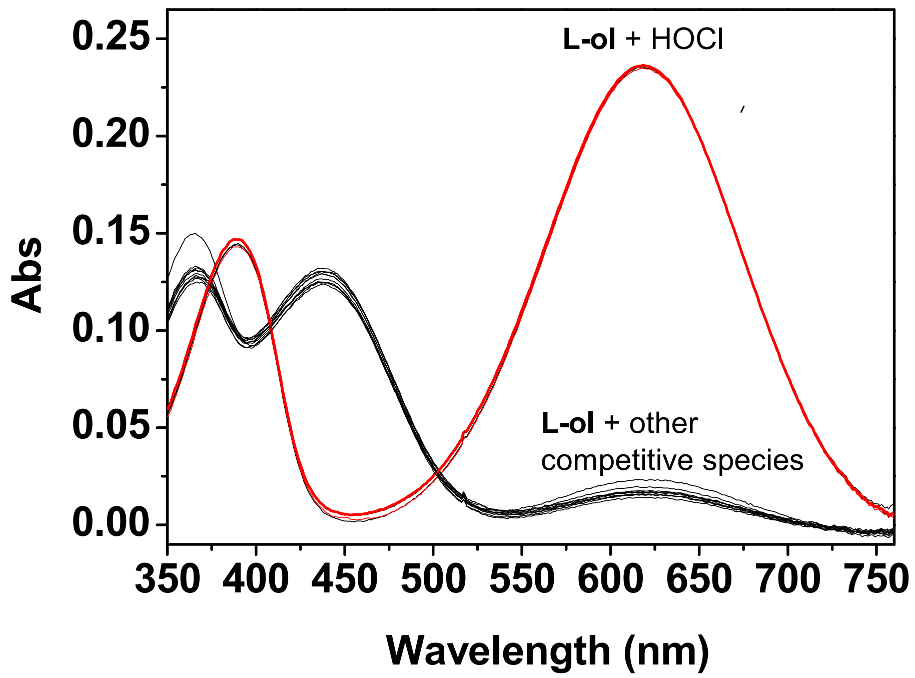

Figure S8. Absorption spectra of **L-ol** (10 μM) in PBS aqueous buffer (DMF: PBS=7:3, 20 mM, pH=7.4) upon addition of various anions (50 μM): Br^-, AcO^-, Cl^-, F^-, HSO_3^-, HSO_4^-, S^{2-}, NO_2^-, NO_3^-, OH^-, PO_4^{2-}, SO_3^{2-}, SO_4^{2-}, HCO_3^-, Pi, PPi, HOCl, $^1O_2$, $ONOO^-$, .OH, H_2O_2, Cys, Hcy and GSH.

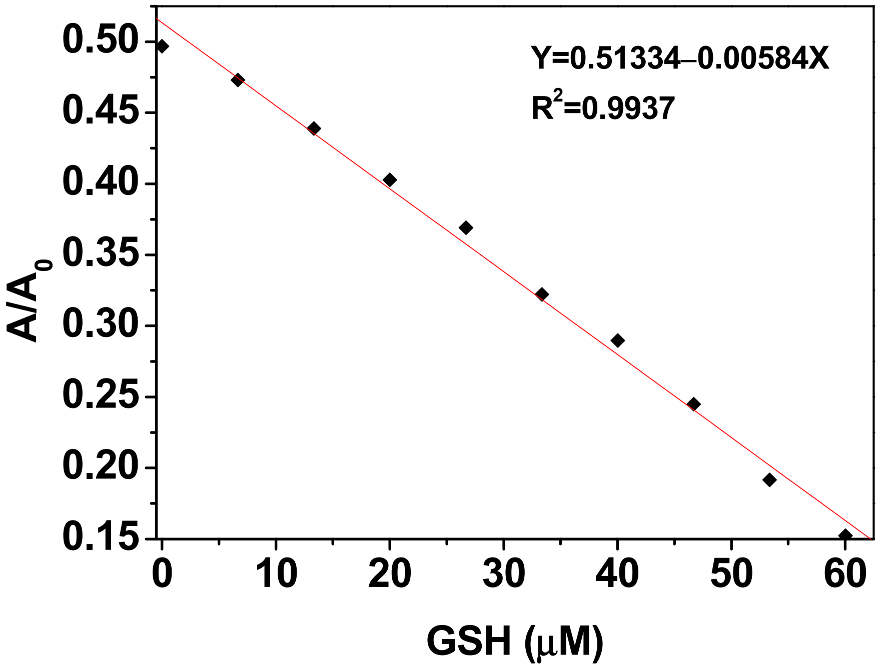

Figure S9. UV-vis absorption spectra changes at 617 nm of **L-one** (2 μM) as function of GSH concentrations (0–6.0 μM).

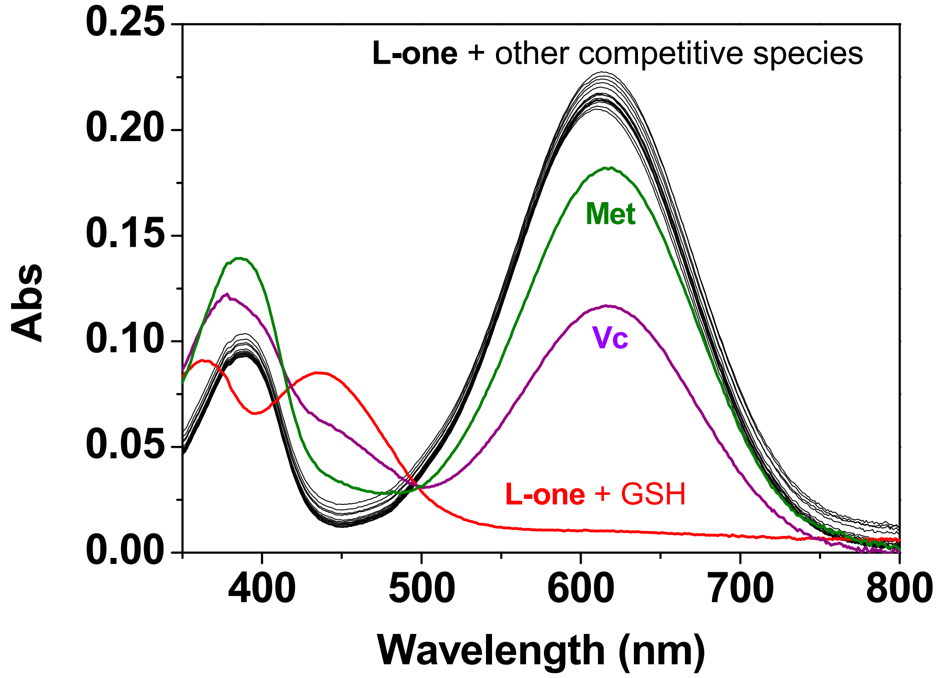

Figure S10. UV-vis absorption spectra of **L-one** (10 μM) in the presence of various analytes (500 μM) in PBS aqueous buffer (DMF:PBS=7:3, 20 mM, pH=7.4): Leu, His, Val, Tyr, Gerl, Try, Phe, Leu, Thr, Lys, Gly, Pro, Ary, Gln, Asn, Asp, Ala, Met, Hcy, Cys, S^{2-}, HSO_4^-, HSO_3^-, SO_4^{2-}, SO_3^{2-}, Vc and GSH.

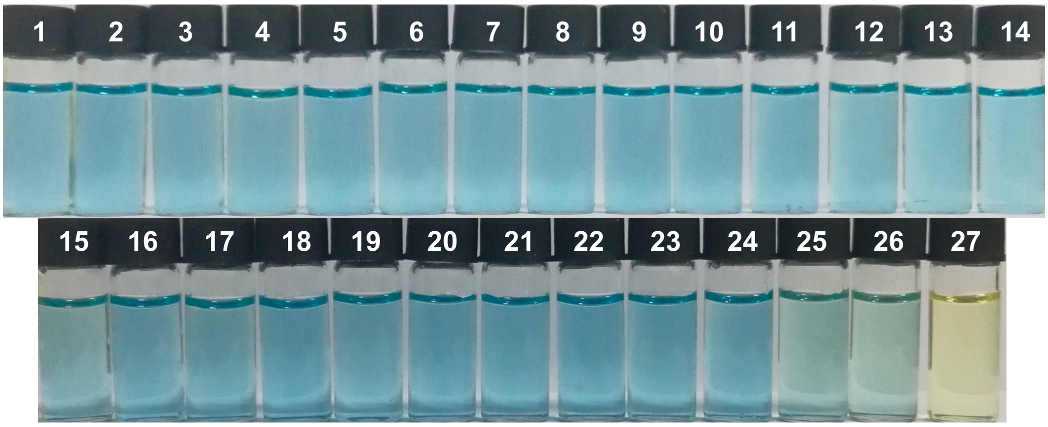

Figure S11. The color changes of **L-one** (10 μM) towards various competitive species (500 μM) in PBS aqueous buffer (DMF:PBS=7:3, 20 mM, pH=7.4). 1. Leu, 2. His, 3. Val, 4. Tyr, 5. Gerl, 6. Try, 7. Phe, 8. Leu, 9. Thr, 10. Lys, 11. Gly, 12. Pro, 13. Ary, 14. Gln, 15. Asn, 16. Asp, 17. Ala, 18. Hcy, 19. Cys, 20. S^{2-}, 21. HSO_4^-, 22. HSO_3^-, 23. SO_4^{2-}, 24. SO_3^{2-}, 25. Met, 26, Cv, 27. GSH.
